# Medicine and creativity in medical psychology

**DOI:** 10.1590/1516-3180.2013.1314638

**Published:** 2013-08-01

**Authors:** Décio Gilberto Natrielli, Mailu Enokibara Silva, Décio Gilberto Natrielli

**Affiliations:** I MD. Attending Psychiatrist, Irmãs Hospitaleiras do Sagrado Coração de Jesus, São Paulo, Brazil.; II MD. Resident, Department of Psychiatry, Centro de Atenção Integrada a Saúde Mental (CAISM), Santa Casa de Misericórdia de São Paulo, São Paulo, Brazil.; III MD. Scientific Coordinator, Multidisciplinary Committee of Medical Psychology, Associação Paulista de Medicina (APM), São Paulo, Brazil.

The art of medicine is related to a precise decision-making process, which has evolved from strict scientific research towards the current practice of evidence-based medicine. Guided by the need to have safe and precise practice, doctors can rely on guidelines and data published in scientific journals to ensure both therapeutic and diagnostic success.

Creativity can be considered to be human beings’ capacity to promote growth of their own and other people’s potentials.^1^ Through cognitive mechanisms acquired from the slow and persistent process of human evolution, the human mind is able to create an amazing field of ideas and models that aim to describe or “translate” nature through mathematics, biology, history, geology, physics and all other kinds of sciences. Use and improvement of these scientific concepts lead towards a wide variety of tools aimed at controlling, transforming and manipulating our world. The complexity of our behaviors and models are closely related to creativity. Regarding medical practice, creativity is unlikely to be limited to scientific research. It has also been correlated with day-to-day approaches towards patients: the so-called doctor-patient relationship.

Creativity can be seen as the possibility of transcending the boundary between a concrete scenario determined by a certain disease (i.e. objective features such as physiological changes) and the subjective world of people undergoing such pathological experiences. Listening to, looking forward to, holding onto and paying attention to all the words and effects or feelings experienced by patients could be a way to practice the use of creativity in medical settings. Despite the simplicity of this practice, it is not widespread today, as can be deduced from the frequent complaints relating to the lack of “humanity” in the current medical environment.

De Masi^2^ argued that within evolution, our brain progressively miniaturized its circuits, multiplied all the functional levels and built internal areas of the cortex. These adaptive mechanisms were fundamental for synthesizing data provided from other brain areas and for developing further mental processes such as abstraction, anticipation and symbolization. Within this scenario, creativity appears as a mechanism for thinking about reality and its relationships. Without creativity, reality becomes cold, flavorless, impoverished, logical, operative and concrete. Without creativity, medicine would be limited to a scientific approach to life; physics and mathematics would be reduced to sciences without philosophy, and history would speak a language differing from literature.

Newton, Bacon, John Stuart Mill and Darwin^2^ followed the inductive method to develop concepts regarding unknown processes. Their strategy was based on a mentalist approach that aimed to reach “higher conclusions” and spread knowledge. In this process, mental work prevails over experimental research.

Developing creativity requires neurocognitive, affective and psychosocial development. We take the view that creativity is an intellectual ability of fundamental relevance regarding interpersonal, working and group relationships. For medicine and doctors, we consider that all of this process (of creativity) plays a major role in clinical practice, and that this can be quite helpful to doctors in three main scenarios:

1. When solving problems and complaints that would otherwise be perceived as less important than the core symptoms of the diseases;
2. Promoting new solutions for conflicts;
3. Strengthening the real and symbolic picture of a doctor as a confident person, open to all the needs of people who we care for.

Indeed, creativity promotes psychological growth among those who are available to stimulate and work out hidden languages (non-verbal or pre-verbal), i.e. different forms of the intrinsic human communication apparatus. This noble task may lead to insights such as making defensive mechanisms adaptable to worldwide demands, or transforming suffering into new positive or adaptive experiences, rather than being a simple dead end. Finally, taking advantage of the evolution model, creativity would transit through this adaptive scenario, thus supporting a “sense of a purpose” and reinforcing the positive affective consequences of guidelines, medical routines and evidence-based medicine.

As mentioned earlier, although this letter has focused on an obvious issue, not all doctors are prepared to deal with the constellation of variables involved in medical practice. We should be aware of this limitation as intrinsic to our practice, and therefore spare some thought for the matter of exploring new languages to describe patients.

Creativity demands energy and is a higher brain function, not an automatic function. It can provide doctors with the ability to perceive different mechanisms underlying what patients tell them, such as their fears, doubts, idealizations or depreciations. Each patient has a unique history, and when physicians formulate diagnosis, they are in fact rewriting their patients’ ideas about the past. Our aim is to attenuate present suffering and prevent future occurrences.
